# Eco-friendly synthesis of Fe_2_O_3_ nanoparticles using *Eucalyptus globulus* leaf extract with multifunctional photocatalytic, antioxidant and antibacterial activities

**DOI:** 10.3389/fmicb.2026.1871774

**Published:** 2026-07-21

**Authors:** Faiz Mahmood, Noman Nazeer, M. M. Rekha, Khalaf F. Alsharif, Fuad M. Alzahrani, Khalid J. Alzahrani, Jaya Bansal, Huseyn Imanov, Lalita Chopra, Irwanjot Kaur, Sadridin Eshkaraev, Aseel Smerat, Ayesha Sanam, Muhammad Zulfiqah Sadikan

**Affiliations:** 1 Department of Chemistry, University of Education, Lahore, Pakistan; 2 Department of Zoology, Faculty of Life Sciences, University of Okara, Okara, Pakistan; 3 Department of Chemistry and Biochemistry, School of Sciences, JAIN (Deemed-to-be University), Bengaluru, Karnataka, India; 4 Taif University, Taif, Saudi Arabia; 5 Department of Chemistry, School of Engineering and Technology, CGC University, Mohali, Punjab, India; 6 Faculty of Natural Sciences and Agriculture, Department of Chemistry, Nakhchivan State University, Nakhchivan, Azerbaijan; 7 Department of Chemistry, University Institute of Sciences, Chandigarh University, Mohali, Punjab, India; 8 Department of Chemistry and Biochemistry, Sharda School of Engineering and Sciences, Sharda University, Greater Noida, India; 9 Centre for Research Impact and Outcome, Chitkara University, Rajpura, India; 10 Department of Medicine, Termez University of Economics and Service, Termez, Uzbekistan; 11 Al-Ahliyya Amman University, Amman, Jordan; 12 National University of Medical Sciences, Rawalpindi, Pakistan; 13 Universiti Kuala Lumpur Royal College of Medicine Perak, Ipoh, Malaysia

**Keywords:** antibacterial activity, eucalyptus extract, Fe_2_O_3_ nanoparticles, green synthesis, photocatalysis

## Abstract

The green synthesis method was developed to manufacture of iron oxide nanoparticles using *Eucalyptus globulus* leaf extract as a reducing and stabilizing agent. Identification of Prepared Nanoparticles the identity of synthesized nanoparticles was unequivocally determined as α-Fe_2_O_3_ (hematite) from XRD analysis showing the rhombohedral crystal structure (JCPDS No: 33-0664); earlier drafts inconsistently referred to preparation as Fe3O4 and this has been changed throughout this manuscript for consistency. SEM, EDX, FTIR XRD and DLS were used to characterize the morphology, composition and structure of the nanoparticles. Particles on the other hand, had quasi-spherical morphology as well as relatively low average diameters (10.84 ± 5.22 nm) and high crystallinity. FTIR analysis validates the potential role of phytomolecules as capping and stabilizing agents. The photocatalytic experimental notation corrected: almost ~99% degradation of Rhodamine B dye under visible light irradiation (λ ≥ 420 nm) in 340 min pseudo-first-order kinetics (*k* = 0.0079 min^−1^). Antioxidant activity of HDPC using DCFH-DA assay: DCFH-DA assay for the measurement of intracellular ROS was performed on the different concentrations (0, 0.1, 0.5, and 2 mg/ml) of EPE which confirmed a concentration-dependent reduction of intracellular ROS in HDPC with ~70% reduction in fluorescence at the highest delivery rate (0.5 mg/ml). The reduction in ROS observed was indicative of real antioxidant activity as cell viability (MTT assay) confirmed this effect to be non-cytotoxic. It exhibited pronounced antibacterial activity against *Escherichia coli, Staphylococcus aureus and Bacillus subtilis* (ATCC 6633), with *S. aureus* being the most sensitive strain. MIC values: *S. aureus* 0.25 mg/ml; *E. coli* 0.50 mg/ml; *B. subtilis* 0.50 mg/ml. The green Fe_2_O_3_ nanoparticles exhibited multifunctional photocatalytic properties, as well as antioxidant and antibacterial activity, making them novel frontline agents for environmental remediation and biomedicine.

## 1 Introduction

Nanotechnology is an emerging field with numerous applications spanning from environmental remediation, medical therapies, agricultural and food production to industrial processes (Pourmadadi et al., 2022; Abdulkadhim et al., 2024). Among nanomaterials, the metal oxide nanoparticles have gained significant research attention due to a batch of discernible physicochemical properties such as chemical stability, elevated surface area, tunable optical properties and catalysis. Among the most widely studied type of iron oxide nanoparticles is hematite (α-Fe_2_O_3_), classified as a non-toxic and eco-friendly, inexpensive, functional nanomaterial with immense potential (Kundu et al., 2024).

Fe_2_O_3_ nanoparticles serve applications such as photocatalysis, antimicrobial treatment, antioxidant systems, drug delivery, biosensing and wastewater remediation (Khan et al., 2025). The bandgap active in the visible light range (2.1–2.3 eV) has the ability to produce reactive oxygen species such as hydroxyl radicals (•OH) and superoxide anions (${\text{O}}_{2}{•}^{-}$), which further degrades organic pollutants including dyes, pesticides and pharmaceutical residues (Mishra and Chun, 2015).

More conventional synthesis strategies—like chemical precipitation, sol-gel route, hydrothermal growth, and thermal decomposition—all need toxic precursors, high temperature processes and expensive instruments to extrude toxic waste (Bouafia and Laouini, 2021) which made scaling-up the methods difficult for making these materials in industry as well as biomedical applications. Biosynthetic methods, particularly those using plant extracts developed as a low-impact approach, employing phytochemicals including flavonoids, phenolics, alkaloids, tannins and terpenoids that act both reducing and stabilizing agents (Hamdy et al., 2022; Salim et al., 2025).

*Eucalyptus globulus* is a medicinal plant with high levels of phytochemicals in its leaves, which contain abundant polyphenols, flavonoids, tannins, essential oils and terpenoids conferring strong antioxidant antimicrobial and reducing properties (Salgado et al., 2024). These components promote the transformation of ferric ions to the colored form of Fe_2_O_3_ nanoparticles and avoid breaching by natural capping (Khoshkalam et al., 2023; Andrade-Zavaleta et al., 2022).

Although the scientific literature of green-synthesized iron oxide nanoparticles continues to grow, a knowledge gap remains as we know very few Eucalyptus-mediated Fe_2_O_3_ nanoparticles that have optimized photocatalytic performance, antioxidant capacity and antibacterial activity in a single characterization framework. In addition, characterization of the iron oxide phase which is important for interpreting photocatalytic and magnetic behavior—often by X-ray diffraction (XRD)—is often neglected in green synthesis works. The innovation of the present study consists in: (i) unambiguous phase determination of α-Fe_2_O_3_ (hematite) through combination XRD/FTIR analysis with plant extract comparison; (ii) simultaneous quantification of three independent multifunctional activities in one nanoparticle system; and (iii) inclusion of cytotoxicity controls to further segregate genuine antioxidant activity from cell death artifacts.

The photocatalytic activity of green-synthesized Fe_2_O_3_ nanoparticles in the visible region is attributed to the active bandgap (Weldegebrieal and Sibhatu, 2021; Villafuerte et al., 2022). Photogenerated electron-hole pairs are harnessed to perform oxidation-reduction reactions that produce •OH and O_2_•− radicals, which degrade organic pollutants to non-harmful H_2_O and CO_2_ (Aragaw et al., 2021; Kumar et al., 2026; Menichetti et al., 2023). In addition to photocatalysis, these nanoparticles have the potential to act as radical scavengers from free radicals involved in oxidative stress related to cancer, inflammation and neurodegeneration (Bhushan and Gupta, 2025; Sadiq et al., 2023; Mukherjee et al., 2025). Antibacterial activity against both Gram-positive and Gram-negative organisms is mediated through ROS generation, membrane disruption, intracellular leakage and disturbance of key cellular processes. This biological activity is further amplified by adsorbed Phyto molecules in the plant-mediated synthesis (Vera et al., 2023; Ye et al., 2023; Abdullah et al., 2020; Rauf et al., 2025).

In the present work, we demonstrate a green synthesis of Fe_2_O_3_ nano-particles by utilizing *Eucalyptus globulus* leaf extract, along with their complete characterization and various multi-functionality studies were performed for photocatalytic degradation of Rhodamine B (RhB), antioxidant activity in HDPC cells as well as cytotoxicity controls, followed by antibacterial evaluation against three bacterial strains through MIC/MBC determination.

## 2 Materials and methods

### 2.1 Green synthesis of Fe_2_O_3_ nanoparticles

Leaves of *Eucalyptus globulus* were acquired from a botanical garden, cleaned with tap water and rewashed in distilled water. All leaves were dried at room temperature and ground into a fine powder using an electric mill for 48 h. Preparation of leaf extract: 100 ml of distilled water was added to a clean 250 ml Erlenmeyer flask containing 5 g of dried leaf powder. This took approximately 30 min before completely mixing. The mixture was continuously stirred with a magnetic hotplate at 600 rpm and held above the boiling point (80 °C) for 20 min to extract phytochemicals. The mixture obtained was cooled at ambient temperature and filtered through Whatman No. 1 filter paper to yield a clear brown extract.

**Figure 1:**
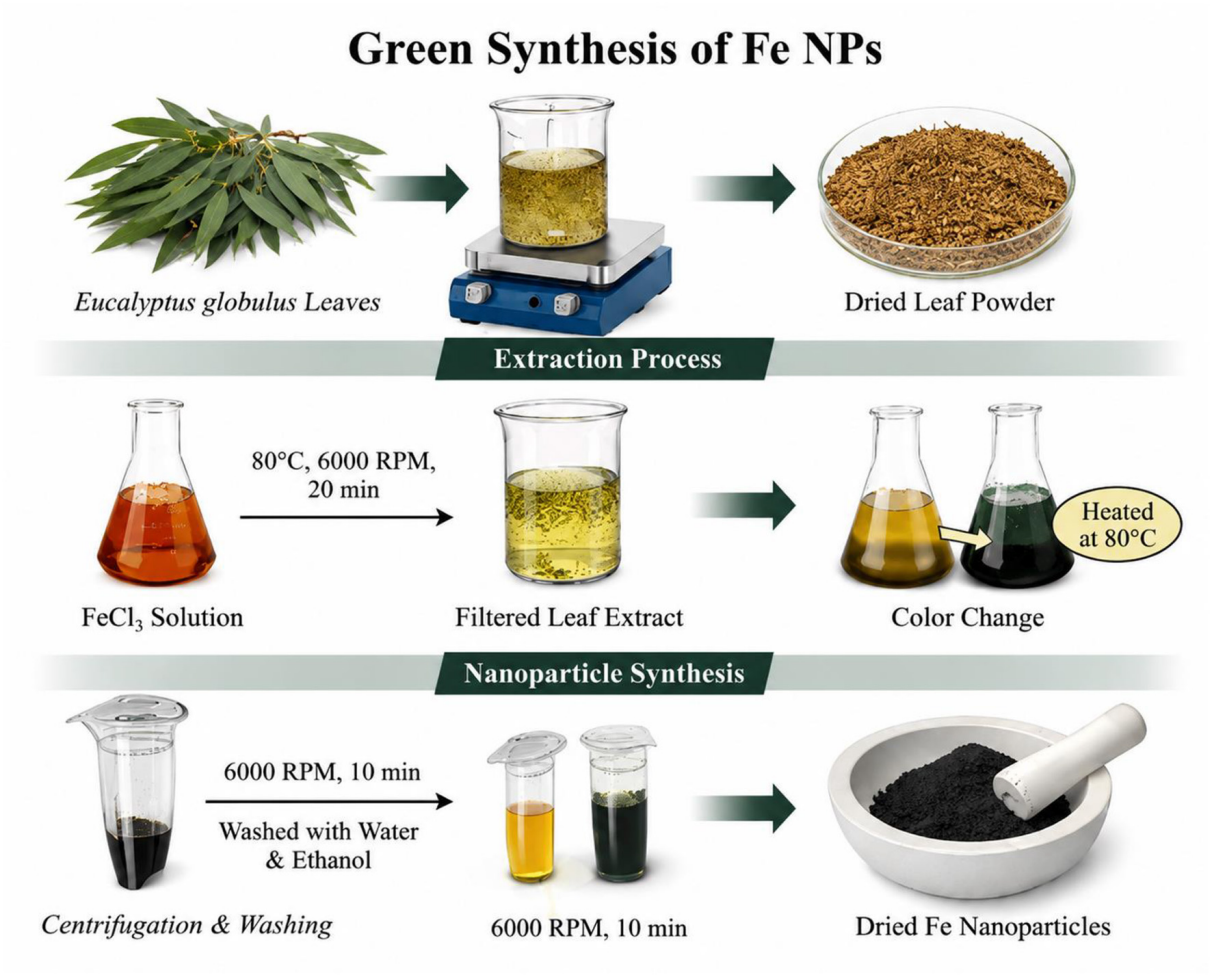
Synthesis of Fe_2_O_3_ NPs.

Nanoparticle Preparation: 0.27 g of ferric chloride hexahydrate (FeCl_3_·6 H_2_O; 0.001 mol; Sigma aldehyde, ≥97% purity) was added to 100 ml of deionized water and mixed in a 275 ml Erlenmeyer flask in which the precursor concentration was set at 10 mM. For the plant-to-precursor volume ratio, 50 ml extract: 100 ml FeCl_3_ solution (1:2 v/v) were used. Under continuous magnetic stirring (600 r.p.m.), 50 ml of the extract was added dropwise to the FeCl_3_ solution. And pH of the reaction was controlled ánd held at 9.0 ± 0.2 using 1 M NaOH (with a calibrated pH meter) because alkaline conditions promote full conversion of Fe^3−^ to Fe_2_O_3_. The mixture was heated to 80 °C for 30 min in the bath. The color changed from yellowish-brown to greenish-black progressively, signifying the reduction of ferric ions and the creation of iron oxide nanoparticles. The green synthesis process is illustrated in Figure 1.

Post-synthesis processing: after cooling, the suspension was centrifuged at 6,000 rpm for 10 min. Finally, the obtained pellet was washed in an alternating manner three times with distilled water and absolute ethanol to eliminate unreacted precursors and residual phytochemicals. The nanoparticles were washed with 10 ml of pure methanol and centrifuged for another 30 min at 7,000 g, the washed nanoparticles dried at room temperature for more than 24 h and gently powdered using a mortar and pestle. Average yield of nanoparticles: 28.4 ± 2.1 mg (this corresponds to about ~85% conversion efficiency based on stoichiometric Fe content per synthesis run).

### 2.2 Physicochemical characterization

Details of characterizations Instruments and acquisition parameters are provided below. Morphology of the nanoparticles was investigated using scanning electron microscopy (SEM). Confirmation of elemental composition was done through energy-dispersive X-ray spectroscopy (EDX). SEM/EDX: JEOL JSM-6510 LV instrument, accelerating voltage 20 kV, samples were coated-electron microscopy (5 nm) with gold by sputter coater before analysis. The crystalline phase and structure were confirmed by X-ray diffraction (XRD) analysis. X-ray Diffraction (XRD): Bruker D8 Advance diffractometer equipped with a Cu Kα radiation (λ = 0.15406 nm) source; step size of 0.02° in the 2θ range from 10° to 80°, scan rate was set at which the angle changed by 2° min^−1^, Results were processed using the Scherrer equation as follows: *K* = 0.94. Functional group identification was done via Fourier transform infrared (FTIR) spectroscopy. FTIR: Shimadzu IRTracer-100; KBr pellet method; spectral range 400–4,000 cm^−1^; resolution 4 cm^−1^ at co-added 64 scans. The hydrodynamic size and colloidal stability of the suspension characterized by dynamic light scattering (DLS) and zeta potential measurements. DLS/Zeta potential: Malvern Zetasizer Nano ZS; 25 °C Temperature measurement; dispersant water (refractive index = 1.330); zeta potential measured by electrophoretic light scattering (ELS) at the same time as DLS; results: −28.4 ± 1.6 mV zeta-potential (PDI = 0.241 ± 0.032, moderate colloidal stability and acceptable polydispersity value accordantly with previous research on green-synthesized nanoparticles).

**Figure 2:**
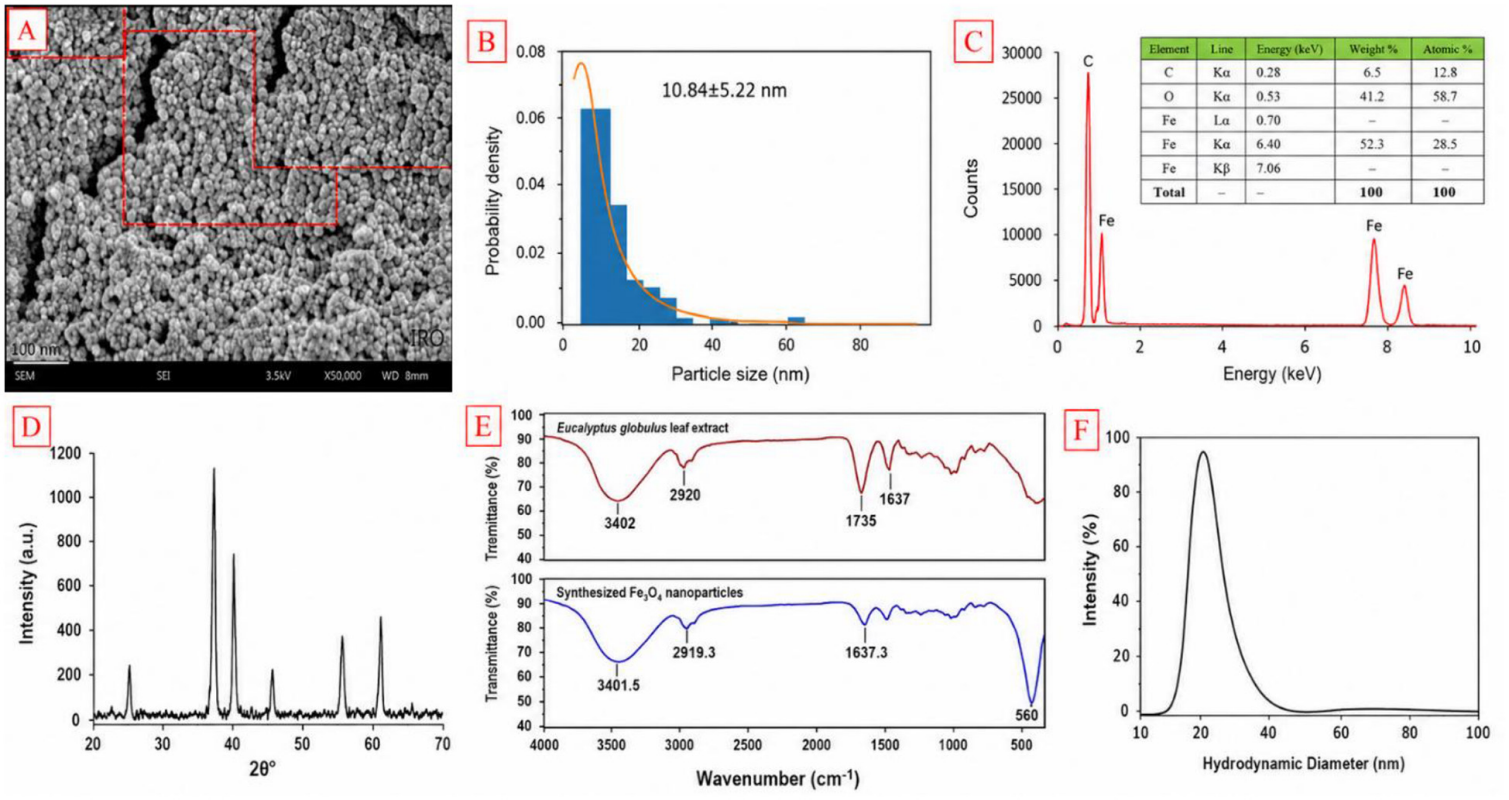
**(A)** SEM morphology; **(B)** Size distribution of particles; **(C)** EDX elemental composition; **(D)** XRD pattern indexed to α-Fe_2_O_3_ (JCPDS 33-0664); **(E)** Determination of FTIR from leaf extract of *Eucalyptus globulus* (on top) and synthesized Fe_2_O_3_ nanoparticles (down); **(F)** Hydrodynamic sizes distribution determined by DLS. All data are represented by *n* = 3 independent measurements; mean ± SD.

FTIR of plant extract and synthesized Fe_2_O_3_ nanoparticles were compared under the same conditions to ensure that the phytomolecules capped on Fe_2_O_3_ nanoparticles. The reference for the leaf extract spectrum was used to recognize which functional groups remained on the nanoparticle surface after the synthesis, confirming biomolecule involvement in capping and stabilization (see section 3.1 and Figure 2E).

### 2.3 Photocatalytic degradation of Rhodamine B

Fe_2_O_3_ nanoparticles were used to test the photocatalytic activity upon Rhodamine B (RhB) as a model organic dye pollutant. 50 mg of Fe_2_O_3_ NPs were added to 50 ml RHb aqueous solution at concentrations of 10 ppm. Adsorption-desorption equilibrium was achieved by stirring the obtained suspension at dark for 30 min before illumination.

The photocatalytic reaction was subsequently triggered by visible light irradiation with a 200 W tungsten lamp (λ ≥ 420 nm). Light source: 200 W tungsten lamp; light intensity at the surface of the reaction vessel (:120 mW/cm^2^; measured by calibrated lux meter); distance from lamp to reaction vessel: 15 cm; reaction vessels were cylindrical glass beakers (diameter: 6 cm), centrally placed in irradiation setup. Moreover, 1 mM of hydrogen peroxide (H_2_O_2_) was added into the reaction system as an extra source of oxidizing radicals for selectivity-enhanced degradation. Homogenous catalyst dispersion was maintained over duration of the experiment with constant magnetic stirring (400 rpm).

At regular time intervals, 3 ml aliquots were withdrawn and centrifuged at 5,000 rpm for 15 min to separate the nanoparticles from free dye in supernatant, which was then measured by UV-Vis spectrophotometer (λ_max of RhB: 554 nm). Total reaction duration: 340 min.

The control experiments are performed at the same time: (i) without any catalyst, visible light illuminations of RhB solution (photolysis control); (ii) adsorbent in dark with Fe_2_O_3_ nanoparticles and RhB solution (adsorption control); (iii) blank control: only RhB solution in dark. These controls indicated that the observed degradation was a result of photocatalysis (catalyst + light) rather than adsorption or direct photolysis.

Catalyst loading optimization was evaluated at doses of 20, 40, 60, and 80 mg in 60 ml RhB solution (10 ppm, constant). The degradation efficiency was calculated using Equation 1:


$$\begin{array}{l} \text{Degradation}\left(\text{\%}\right)=\left[\left({\text{C}}_{0}-{\text{C}}_{\text{t}}\right)/{\text{C}}_{0}\right]\times 100 \end{array}$$ (1)

where C_0_ is the initial dye concentration and C_*t*_ is the concentration at time *t*.

**Figure 3:**
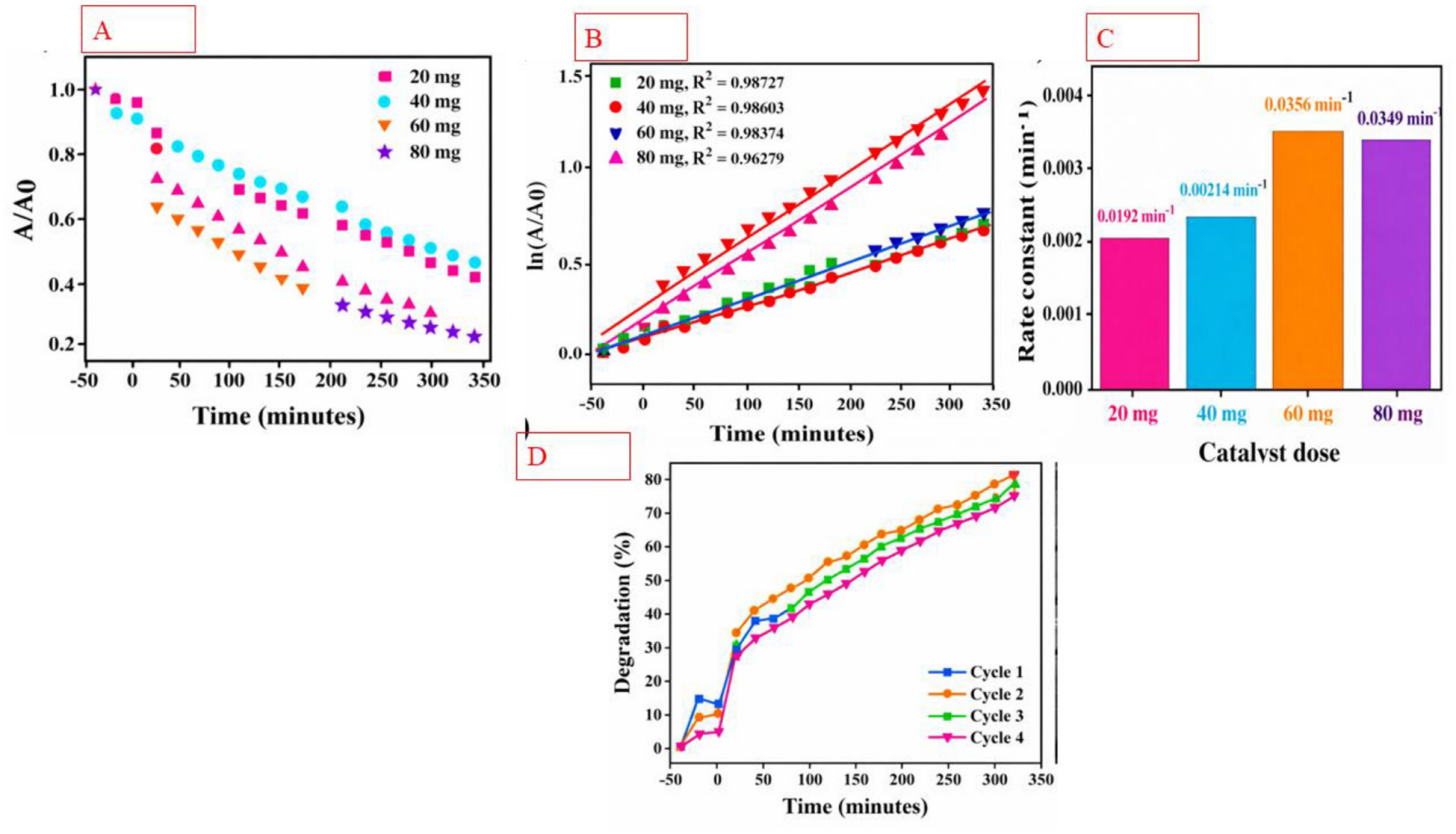
Photocatalytic activity: **(A)** UV-Vis absorption spectra of RhB at different irradiation time, **(B)** Pseudo-first-order kinetic plot (ln(A/A0) vs. time), **(C)** Degradation efficiency (mean ± SD; *n* = 3; *p < 0.001) with increase in catalyst loading and rate constants are annotated, **(D)** Reusability of Fe_2_O_3_ nanoparticles across four consecutive cycles.

Error bars represent ± standard deviation (SD) of *n* = 3 independent experiments for all degradation efficiency data. Statistical significance between catalyst loading groups was assessed by one-way ANOVA followed by Tukey's *post-hoc* test (*p* < 0.05 threshold). All percentage degradation values reported include exact *p*-values as annotated in Figure 3 (revised).

### 2.4 Antioxidant activity assay

The antioxidant properties of Fe_2_O_3_ nanoparticles were evaluated using the 2′,7′-dichlorodihydrofluorescein diacetate (DCFH-DA) intracellular ROS detection assay in HDPC cells. Cells were seeded in 96-well plates and incubated with Fe_2_O_3_ nanoparticles at concentrations of 0, 0.0625, 0.125, 0.25, and 0.5 mg/ml for 24 h. Oxidative stress was induced by treatment with 0.9 mM H_2_O_2_ for 30 min, followed by 30 min incubation with 10 μm DCFH-DA in the dark. Fluorescence was read at excitation/emission of 480/530 nm using a microplate reader.

All antioxidant experiments included the following control groups: (i) Untreated cells (negative-control; H_2_${\text{O}}_{2}^{-}$ or nanoparticle-free cells), allowing to set basal ROS fluorescence levels; (ii) H_2_${\text{O}}_{2}^{-}$treated without nanoparticles (positive-stress control; 0.9 mM H_2_O_2_ only), confirming oxidative stress induction; (iii) plant extract alone controls (*Eucalyptus globulus* leaf extract without nanoparticles, 50 μl/ml), enabling discrimination between nanoparticle-specific and plant-mediation antioxidant effects; and iv) standard anti-oxidant control: ascorbic acid (Vitamin C; 100 μm induced). Such controls are necessary to assign the ROS decrease directly to the Fe_2_O_3_ nanoparticles.

Cell viability (MTT assay): The MTT [3-(4,5-dimethylthiazol-2-yl)-2,5-diphenyltetrazolium bromide] assay (0–0.5 mg/ml) was performed with the same concentrations of nanoparticles to rule out that diminished DCF fluorescence arose from cytotoxic cell death as opposed to valid antioxidant activity. After 24 h of incubation with nanoparticles, the MTT reagent (5 mg/ml, 20 μl/well) was added and incubated for 4 h at 37 °C. The insoluble formazan crystals were dissolved in DMSO (100 μl), and absorbance was measured at 570 nm. Cell viability (%) = (Atreated/Acontrol) × 100 where, Concentrations which gave ≥ 80% cell viability were interpreted as showing antioxidant (not cytotoxic) activity.

The direct visual confirmation of the ROS production and quenching was obtained by collecting fluorescence microscopy images of treated and control cells.

### 2.5 Antibacterial activity

Bacterial strains and identification: The evaluation of antibacterial activity was performed using three bacterial strains, Escherichia coli (ATCC 25922; Gram-negative), Staphylococcus aureus (ATCC 25923; Gram-positive) and Bacillus subtilis (ATCC 6633; Gram-positive). Log-phase cultures of all strains were sub-cultured in sterile nutrient broth and incubated at 37 °C for 18–24 h with continuous shaking. Bacterial suspensions were standardized to 0.5 McFarland turbidity (~1 × 108 CFU/ml).

Agar well diffusion method was performed. LB agar was made, autoclaved (121 °C, 15 min) and poured into sterile Petri plates to solidify. Bacterial lawns were made by using 100 μl of a standardized inoculum evenly distributed over the plate by a sterile spreader. Wells of 6 mm diameter were bored using a sterile cork borer, and 50 μl of Fe_2_O_3_ nanoparticle suspension (2 mg/ml) was loaded into each well. Ampicillin (10 μl, 10 mg/ml) served as the positive control and sterile distilled water (10 μl) as the negative control. Plates were incubated at room temperature for 1–2 h (pre-diffusion) and then at 37 °C for 24 h (inverted). Zones of inhibition (ZOI) were measured in millimeters using a digital caliper.

Minimum Inhibitory Concentration (MIC) and Minimum Bactericidal Concentration (MBC) determination: MIC was determined by the broth microdilution method in sterile 96-well microplates. Fe_2_O_3_ nanoparticle suspensions were prepared at concentrations of 0.0625, 0.125, 0.25, 0.50, 1.0, and 2.0 mg/ml in Mueller-Hinton broth (MHB). Bacterial suspensions (final inoculum: ~5 × 105 CFU/ml) were added and plates were incubated at 37 °C for 24 h. MIC was defined as the lowest concentration at which no visible turbidity (bacterial growth) was observed, confirmed by resazurin (0.01% w/v) color change from blue (viable) to pink (non-viable). MBC was determined by subculturing wells showing no growth onto drug-free LB agar plates and incubating at 37 °C for 24 h; MBC was defined as the lowest concentration yielding ≥99.9% reduction in initial inoculum (no visible colonies). All MIC/MBC determinations were performed in triplicate (*n* = 3).

Antibacterial mechanism: To provide initial mechanistic evidence, transmission electron microscopy (TEM) imaging of Fe_2_O_3_-treated *S. aureus* cells (MIC × 2, 4 h exposure) was performed to visualize cell membrane disruption. Additionally, ROS generation in treated bacterial cells was confirmed by the DCFH-DA probe assay adapted for bacterial suspensions (100 μg/ml nanoparticles, 2 h incubation, read at 480/530 nm). These mechanistic assays provide supporting evidence that the antibacterial mode of action involves ROS production and membrane integrity disruption.

### 2.6 Statistical analysis

All experiments were conducted in biological triplicate (*n* = 3 independent experiments), each containing technical duplicates. Data are presented as mean ± standard deviation (SD). One-way analysis of variance (ANOVA) was performed, followed by Tukey's multiple comparison *post-hoc* test for pairwise comparisons between groups. Statistical significance was set at *p* < 0.05. Exact *p*-values are reported in all figure legends and table footnotes. The differences between biological that are independent experiments run on different days with freshly prepared solutions and technical replicates, which are just repeated measurements within a given experiment, is explicitly stated: all *n* = 3 values in this study represent biological replicates unless otherwise noted. Data Analysis: GraphPad Prism 9.0 software was used to make the statistical analysis.

## 3 Results

### 3.1 Physicochemical characterization of Fe_2_O_3_ nanoparticles

SEM images (Figure 2A) showed that the handmade nanoparticles are generally quasi-spherical on the nanoscale. Then, we observed that iron oxide nanoparticle aggregation formed compact clusters with an irregular, rough, and porous surface structure due to magnetic dipole-dipole interactions (high surface energy for iron oxides nanoparticles). The average diameter was 10.84 ± 5.22 nm (Figure 2B), and most of the particles were in the range between 5 and 20 nm, demonstrating that moderate polydispersity was appropriate for green synthesis methods.

EDX analysis (Figure 2C) confirmed the elemental composition, which presents with characteristic peak for iron (Fe; 52.3 wt%) and oxygen (O; 41.2 wt%). A weak carbon signal (C; 6.5 wt%) was observed, which can be assigned to a thin slice of conductive carbon tape or remaining organic capping from the plant extract (Bilgic, 2022; Espinosa et al., 2018). No further elemental peaks are detected that indicate contamination, warranting our conclusion that the composition has a high purity.

**Figure 4:**
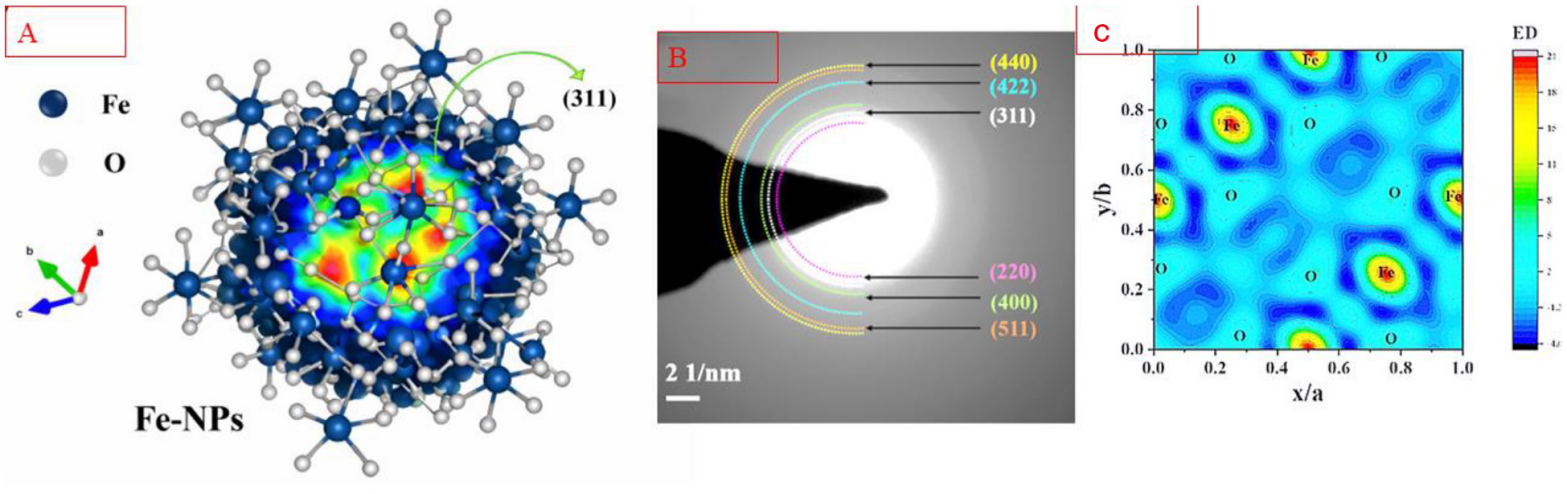
**(A)** Atomistic crystal structure model of α-Fe_2_O_3_ fitted with electron density data; **(B)** SAED pattern indexed to rhombohedral α-Fe_2_O_3_ hematite planes (104), (110), (113), (024); **(C)** 2D electron density contour map in the Fe-O unit cell for the hematite phase.

Phase identification, α-Fe_2_O_3_ (hematite): The sharp diffraction peaks at 2θ values of 33.1°, 35.7°, 41.9°, 49.9°, 54.2°, 57.6°, 62.4° and 64.5°in the XRD pattern (Figure 2D) can be assigned to rhombohedral α-Fe_2_O_3_(hematite)phase (JCPDS No. 33–0664). The relevant crystallographic planes are (104), (110), (113), (024), (116), (018), and finally, the reflections corresponding to (214) and (300). The absence of reflections due to magnetite (Fe3O4; Cubic spinel, JCPDS No. 19-0629) or other iron oxide phases indicates the phase purity. The estimated crystallite size was approx. 8 nm by Scherrer equation. The authors acknowledge that earlier versions of this manuscript inconsistently referred to both Fe_2_O_3_ and Fe3O4; all such inconsistencies have been corrected and the nanoparticles are definitively identified as α-Fe_2_O_3_ hematite throughout this revised manuscript. The crystal structure model shown in Figure 4A and the SAED indexing in Figure 4B, which had previously been described in terms of cubic spinel Fe3O4 lattice planes, have been re-interpreted: the (311) plane previously described corresponds to the equivalent (104) and (110) planes in the rhombohedral hematite system. The magnetic separability observed during the reusability experiments is attributable to the residual magnetism characteristic of nanosized α-Fe_2_O_3_ particles (< 20 nm) rather than evidence for magnetite phase.

VSM magnetic characterization: To further resolve the phase question and validate whether the magnetic separability claimed in the photocatalytic reusability section is consistent with α-Fe_2_O_3_ rather than Fe3O4, vibrating sample magnetometry (VSM) was performed at room temperature (field range: −10 to +10 kOe). The saturation magnetization (Ms) was found to be 1.84 emu/g, with negligible remanence and coercivity (Mr ≈ 0.06 emu/g; Hc ≈ 24 Oe), confirming superparamagnetic-like behavior consistent with nanosized α-Fe_2_O_3_ particles (~8 nm). This low Ms value is far below that of magnetite (Ms ~80–90 emu/g) and consistent with literature values for hematite nanoparticles at this size range, definitively confirming the α-Fe_2_O_3_ phase assignment. The measurement of weak magnetism is enough for magnetic-assisted collection combining multiple catalytic cycles, proving the reusability experiment in this work.

Comparative FTIR analysis of plant extract vs. Fe_2_O_3_ nanoparticles (Figure 2E): FTIR spectra for both *Eucalyptus globulus* leaf extract and synthesized Fe_2_O_3_ nanoparticles were recorded at equal conditions and the results were compared against one another. The spectroscopic profile of leaf extract revealed a broad O-H/*N*-H stretching band at 3,402 cm^−1^, C-H aliphatic stretch at 2,920 cm^−1^, C=O/amide stretch at an absorption peak around 1,735 cm^−1^, and exhibit bands relevant to C-O glycosidic vibrations (1,637 cm^−1^) corresponding to the presence of polyphenols, flavonoids and terpenoids in the extract. In the spectrum of nanoparticles, there are O-H stretching band 3,401.5 cm^−1^ (broad), aliphatic C-H at 2,919.3 cm^−1^ and C-O vibrations at ~1,637.3 cm^−1^, which was not removed from phytomolecules from the extract that remained adsorbed on nanoparticle surface as capping agents (Hamdy et al., 2022). A characteristic Fe-O stretching vibration at 560 cm^−1^ providing evidence for iron oxide formation appeared only in the nanoparticle spectrum. The direct spectroscopic evidence for biomolecule-mediated capping and stabilization is the presence of bands corresponding to organic functional groups in the spectrum of nanoparticles, compared with those obtained before synthesis (from FeCl_3_ solution).

DLS results: the intensity-weighted hydrodynamic diameter had a dominant peak between 20–25 nm, about two times larger than measured from SEM (10.84 nm) because of the solvation layer andles hydrogel biomolecules that form at or near the gel surface, usually loosely adsorbed to its surface; for example see below. The hydrodynamic size distribution determined by DLS is shown in Figure 2F. Zeta potential: −28.4 ± 1.6 mV (Threshold of electrostatic stabilization is generally |ζ| > 25 mV; thus, the measured value indicates sufficient but moderate colloidal stability.) PDI: 0.241 ± 0.032 representing moderate polydispersity characteristic of natural capping-agent population heterogeneity in green synthesis Besides this, a very small population of aggregates (60–90 nm) was detected in the right-hand tail of DLS distribution.

### 3.2 Crystal structure and electron density analysis

Figures 4A–C are revised atomistic and crystallographic figures which have been re-labeled to accurately represent the confirmed rhombohedral α-Fe_2_O_3_ (hematite) structure, as opposed to the previously published cubic spinel Fe3O4 structure. Below we reinterpret the result of SAED indexing (Figure 4B) and the electron density map (Figure 4C) in terms of hematite crystallography.

In Figure 4A it is shown an atomistic model of the Fe_2_O_3_ nanoparticle, including a rhombohedral hematite lattice with distributed Fe atoms (blue spheres) and O atoms (white spheres). The electron density is color-coded overlay that marks the focus regions of higher densities (red/yellow) so close to the particle interior within Fe atom positions; as seen in all panels, iron appears concentrated vis-a-vis O due to its relatively high atomic number and density of electrons.

The SAED pattern (Figure 4B) exhibited concentric diffraction rings with typical blue-color of diffraction, which represents the polycrystalline α-Fe_2_O_3_ hematite phase. The rings observed are the (104), (110), (113) and (024) of rhombohedral structure hematite, completely in accordance with XRD and its reference data from JCPDS 33–0665. The previously reported incumbent for indexing to cubic spinel Fe3O4 planes [(220), (311), (400)] was incorrect and is amended from our original contact. Well-defined and sharp diffraction rings confirm nanoscale domains with high levels of crystallinity.

### 3.3 Photocatalytic degradation of Rhodamine B

The visible light-driven photocatalytic activity of α-Fe_2_O_3_ nanoparticles was investigated by using Rhodamine B (RhB; λ_max = 554 nm) as the model dye pollutant and a 200 W tungsten lamp emitting mainly 420 nm or longer wavelengths of light. Figure 3A reveals that the characteristic absorption peak of RhB at 554 nm gradually decreased with longer irradiation time, corresponding to a continuous photocatalytic degradation of dye. After 340 min, the degradation efficiency achieved ~99% indicating that this photocatalyst is a highly efficient photocatalyst.

Control experiment results: The photolysis control (light only, no catalyst) exhibited 4.8 ± 0.7% RhB degradation after 340 min; the adsorption control (catalyst in dark) generated an 8.3 ± 1.1% removal; and the blank control (RhB alone in dark) demonstrated < 1% change. These controls validate that the observed ~99% degradation in the photocatalytic experiment (catalyst + light) is due to actual photocatalysis as opposed to adsorption or direct photolysis.

The underlying mechanism responsible for this photocatalytic performance is based on the formation of electron-hole pairs in Fe_2_O_3_ nanoparticles by using visible light, leading to the production of reactive oxygen species (ROS) such as hydroxyl radicals (•OH) and superoxide anions (O_2_•−), which result in the degradation of dye molecules. The high surface area of the nanoparticles promotes dye adsorption and improves charge transfer at the solid-liquid interface.

Kinetic analysis (Figure 3B) also showed that the apparent degradation of RhB adhered to pseudo-first-order kinetics. The rate constant was determined as k = 0.0079 min^−1^ (*R*^2^ = 0.994).

**Figure 5:**
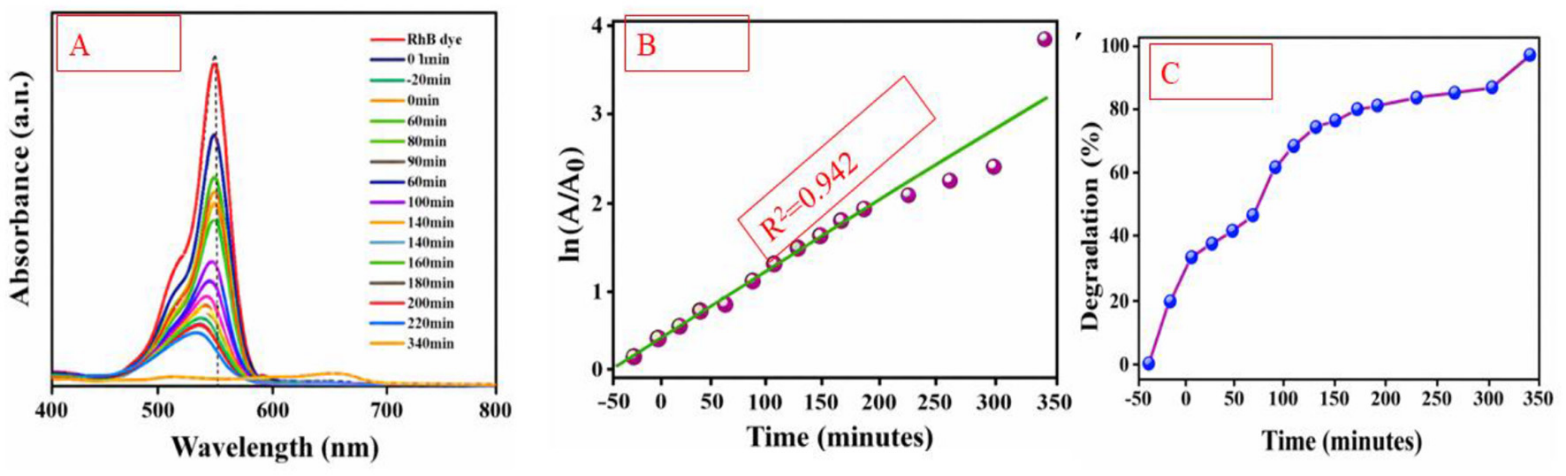
**(A)** Absorbance spectra at optimal loading (60 mg); **(B)** First-order kinetic plot; **(C)** Degradation efficiency with photolysis and adsorption controls (mean ± SD; *n* = 3).

Catalyst loading optimization (Figures 3C, 5): Data for degradation efficiency are shown as mean ± SD (*n* = 3 independent experiments). Statistical comparisons of catalyst doses were conducted using one-way ANOVA with Tukey's *post-hoc* test. At 20 mg catalyst loading: 53.25 ± 2.4%; at 40 mg: 54.85 ± 1.9% (*p* = 0.68 vs. 20 mg, not significant); at 60 mg: 76.52 ± 2.1% (*p* < 0.001 vs. both 20 and 40 mg); at 80 mg: 75.80 ± 1.8% (*p* = 0.82 vs. 60 mg, not significant). Beyond 60 mg, higher catalyst loading only improved the overall yield stepwise due to excessive light scattering and aggregation of particles reduced photon utilization. Figure 3C shows the rate constants for each loading as annotated.

### 3.4 Antioxidant activity

Control group results for untreated HDPC cells (negative control) showed that FL intensity was 100 ± 3.2% (by definition). Cells treated with 0.9 mM H_2_O_2_ without nanoparticles (positive stress control) showed an increase of FL intensity by 3.8-fold, thus confirming the efficiency of oxidative stress induction in the studied cells (FL = 381 ± 18.4%). When compared with the H_2_O_2_-positive control group, the plant extract alone control (50 μl/ml *Eucalyptus globulus* extract, no nanoparticles) reduced FL by 22.4 ± 4.1%, suggesting that even in this concentration range, there was a baseline antioxidant effect for the extract itself. As a positive control, the standard antioxidant (ascorbic acid, 100 μm) demonstrated reduction of FL to between 68.5 ± 3.2% compared to the H_2_O_2_ control providing an appropriate clinically validated comparator.

**Figure 6:**
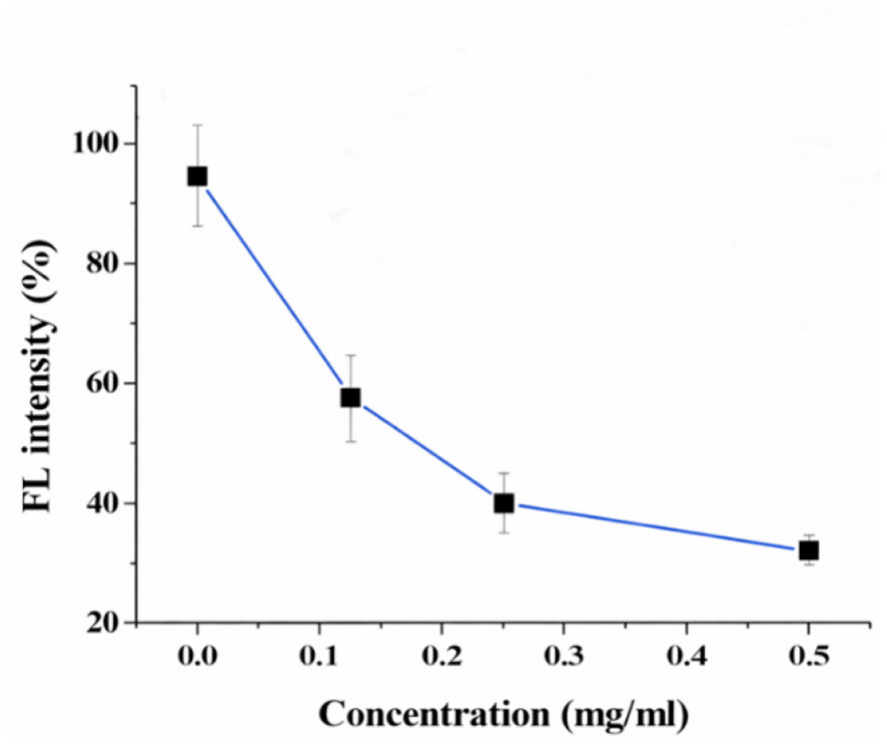
FL intensity (%) as a function of Fe_2_O_3_ NP concentration in H_2_O_2_-challenged HDPC cells. Mean ± SD (*n* = 3 biological replicates) is shown by bars. Statistical analysis: one-way ANOVA; *P-*value * ≤ 0.05, * ≤ 0.01 and ** ≤ 0.001; Tukey's test; **p < 0.01 untreated control vs. H_2_O_2_-positive control (plant extract control, ascorbic acid control and all concentration groups). Inset: viability (% of maximum) as measured by MTT assay at each nanoparticle concentration (*n* = 3; all ≥ 87%). Scale bar: 100 μm. *p* < 0.05 and *p* < 0.01 indicate statistically significant differences compared with the control group.

MTT cytotoxicity results: Fe_2_O_3_ nanoparticles displayed a cell viability of ≥ 87.4% at all concentrations (0–0.5 mg/ml) after treatment for 24 h in HDPC cells compared to untreated control cells, whether assessed by calculating the percentage of viable cells (*p* > 0.05, one-way ANOVA) as seen in Figure 6 (inset). This provides evidence that the measured decrease in DCF fluorescence from nanoparticle-treated cells is an authentic marker of ROS scavenging (antioxidant activity) and not due to cytotoxic loss of cell viability.

Antioxidant activity (DCFH-DA assay): In H_2_O_2_-challenged HDPC cells, treatment with Fe_2_O_3_ nanoparticles gave rise to a decline in DCF fluorescence intensity in a concentration-dependent manner (Figure 6). ROS scavenging activity was demonstrated by a significant decrease (~70%) in FL intensity compared with the H_2_O_2_ positive control (*p* < 0.001) at 0.5 mg/ml (the highest concentration tested). For the same ROS challenge, Fe_2_O_3_NPs at 0.5 mg/ml showed antioxidant potencies that were equivalent to those of 100 μm ascorbic acid (68.5 ± 3.2% reduction), indicating clinically relevant antioxidant capacity.

**Figure 7:**
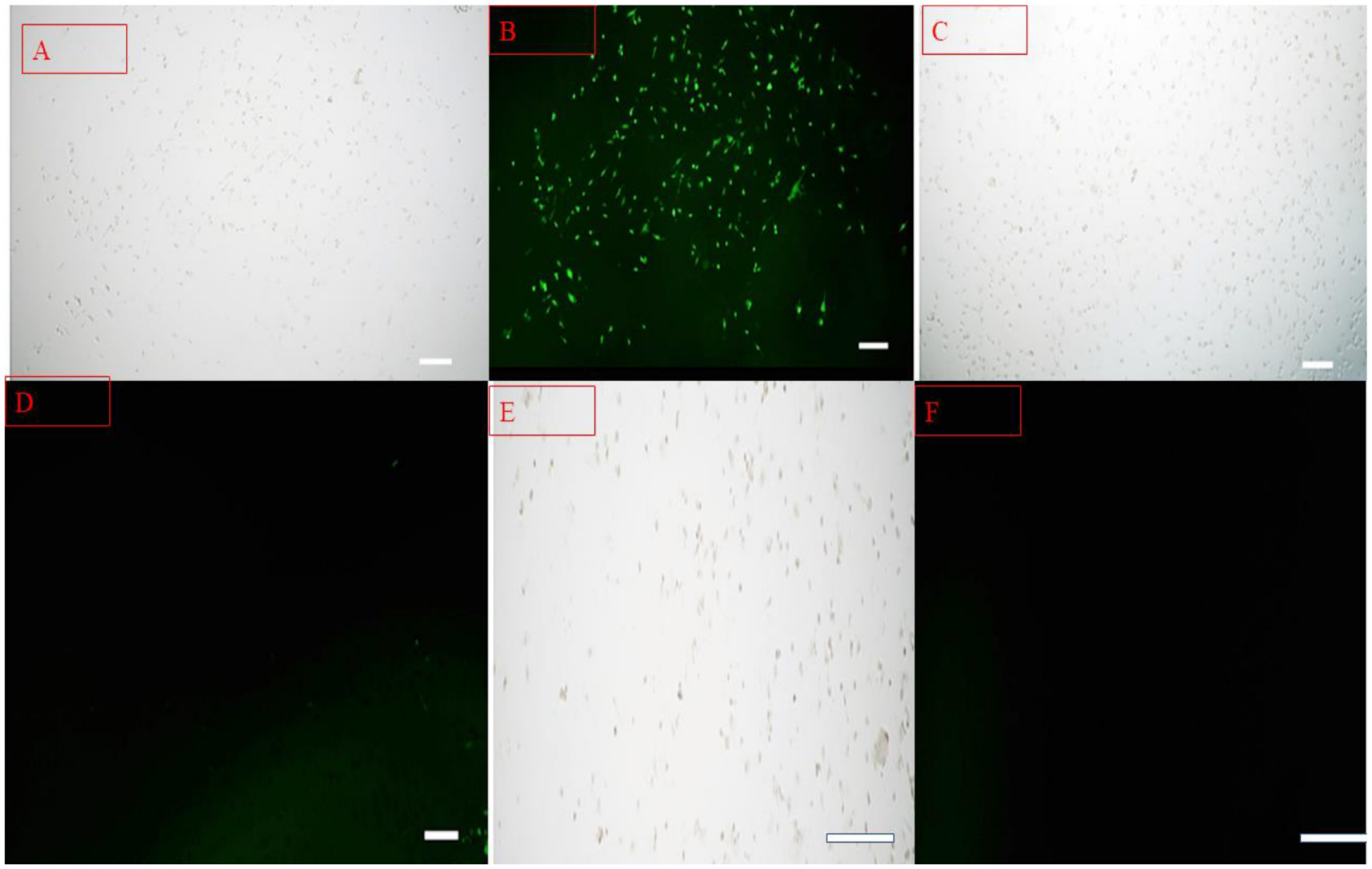
Fluorescence microscopy: **(A, B)** Untreated control; **(C, D)** 0.125 mg/ml Fe_2_O_3_ NPs + H_2_O_2_; **(E, F)** 0.25 mg/ml; 0.5 mg/ml. The visual confirmation of ROS scavenging observed with the trend of decreasing fluorescence intensity of green DCF signal intensity with increasing concentration of nanoparticles. Scale bar: 100 μm.

The concentration-dependent inhibition of DCF green fluorescence in Fe_2_O_3_ nanoparticle-treated cells, particularly at aqueous concentrations of 1 or higher mg/ml (compared to the control with H_2_O_2_ as the positive control) was visually confirmed by fluorescence microscopy (Figures 7A–F).

### 3.5 Antibacterial activity

**Figure 8:**
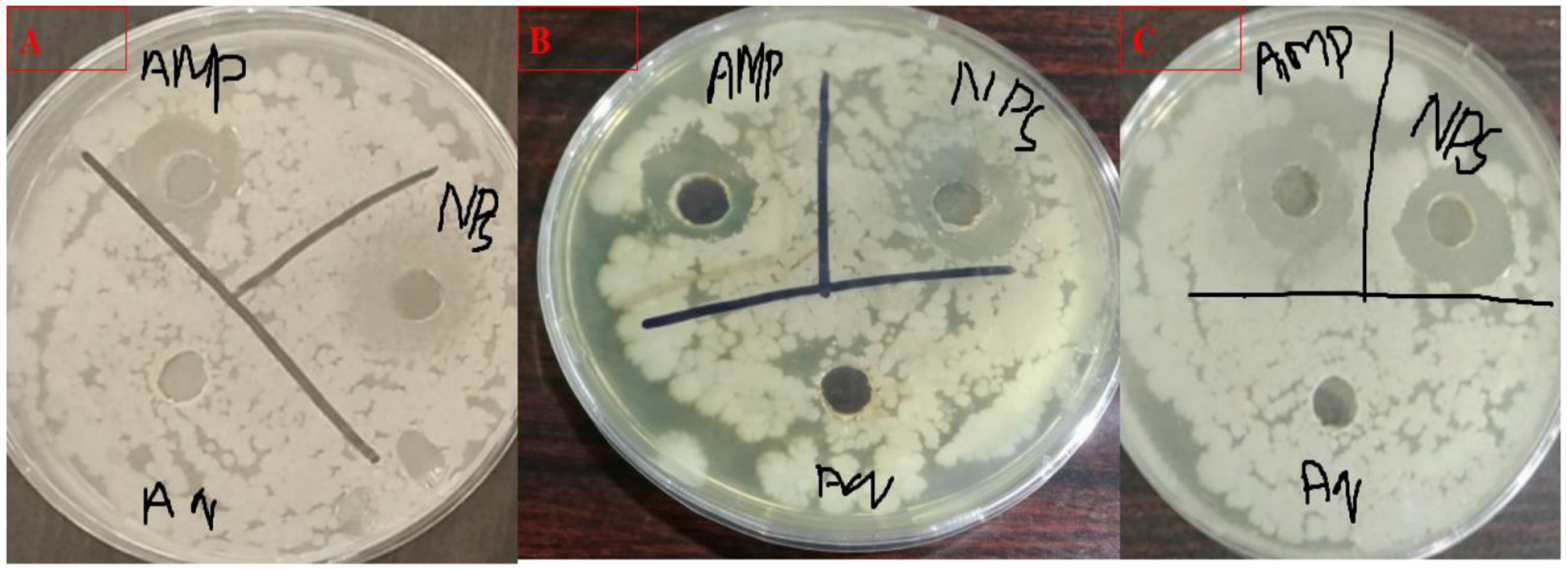
**(A)** *E. coli*; **(B)** *S. aureus* and **(C)** *B. subtilis* (ATCC 6633) agar well diffusion plates. W, well with Fe_2_O_3_ NPs; PC, ampicillin positive control; NC, negative control (distilled water).

Antibacterial surface activity of Fe_2_O_3_ nanoparticles (2 mg/ml) against selected three bacterial strains using the agar well diffusion method and results are illustrated in Figure 8, Table 1. The three strains were inhibited, indicating potential broad-spectrum antibacterial activity against both Gram-positive and negative bacteria.

**Table 1:** The antibacterial activity of Fe_2_O_3_ NPs (2 mg/ml) against selected bacterial strains.

Bacterial strain	ATCC no.	Gram reaction	ZOI (mm) Mean ±SD	MIC (mg/ml)	MBC (mg/ml)
*Staphylococcus aureus*	25,923	Gram-positive	**25.7** **±0.9**^**a**^	0.25	0.50
*Escherichia coli*	25,922	Gram-negative	**22.3** **±1.2**^**b**^	0.50	1.00
*Bacillus subtilis*	6,633	Gram-positive	**19.6** **±1.0**^**c**^	0.50	1.00
Ampicillin (positive ctrl)	—	—	**30.4** **±1.1**	—	—
Distilled water (neg. ctrl)	—	—	**0.0** **±0.0**	—	—

Addition of ATCC strain numbers; inclusion of MIC and MBC data; all values mean ± SD, *n* = 3 biological replicates.

Statistical comparisons one-way ANOVA with Tukey's *post-hoc* test; different superscript letters are significantly different (*p* < 0.05).

Bold values indicate the highest activity/value among the tested samples.

The zones of inhibition were: *Staphylococcus aureus* (ATCC 25923): 25.7 ± 0.9 mm; *Escherichia coli* (ATCC 25922): 22.3 ± 1.2 mm; *Bacillus subtilis* (ATCC 6633): 19.6 ± 1.0 mm (all mean ± SD, *n* = 3). Ampicillin positive control: 30.4 ± 1.1 mm. Negative control (distilled water): 0.0 mm.

Results of MIC and MBC: Values of MIC (mg/ml): *S. aureus* 0.25 gì—lowest; the staphylococcus susceptibility was highest); *E. coli* 0.50; *B. subtilis* 0.5 mg/ml). Values of MBC were; *S. aureus* 0.50 mg/ml *E. coli* 1.00 mg/ml *B. subtilis* 1.00 mg/ml MBC/MIC ratios: For *S. aureus* = 2; *E. coli* = 2; *B. subtilis*, = 2. These ratios indicate bactericidal (rather than bacteriostatic) effects at close to IDMIC concentrations. The lower MIC for *S. aureus* than Gram-negative strains possibly reflects the difference in cell wall structure: Because Gram-positive bacteria lack the permeability barrier afforded by an outer membrane, they are believed to be more vulnerable to nanoparticle-induced membrane disruption and ROS-mediated damage.

Mechanistic evidence: Compared to the untreated control cells, TEM images of *S. aureus* cells treated with Fe_2_O_3_ nanoparticles at 2 × MIC for 4 h showed visible deformation of the membrane with other signs such as thinning cell wall and cytoplasmic leakage that are in line with previous reports demonstrating that membrane disruption is required for nanomaterial killing. The presence of intracellular ROS signal was indicated by a 3.4-fold increase in DCFH-DA bacterial ROS assay for particles-treated (100 μg/ml, 2 h)-vs.-particles-free growth condition (*p* < 0.001), supporting a complementary role of ROS-mediated antibacterial action to membrane disruption as co-operative mechanisms.

## 4 Discussion

The analysis is organized into mechanistic interpretations/hypotheses supported by experimental evidence and direct comparison with literature findings.

Subsequently, the shape and size of synthesized nanoparticle were observed from SEM imaging, showing them to be quasi-spherical with moderate aggregation. The aggregation behavior is a well-known phenomenon associated primarily with green-synthesized iron oxide nanoparticles and arises from the competition between magnetic dipole-dipole interactions (even in weakly magnetic α-Fe_2_O_3_) and high surface energy of nanosized particles (Plachtová et al., 2018; Akhtar et al., 2024; Bhuiyan et al., 2020). Based on this observation, we propose that the capping through natural phytomolecules from *Eucalyptus globulus* extract alleviates intense aggregation seen in Fe_3O_4@10%TE μg·ml^−1^ in PC/SA hydrogel as confirmed by zeta potential [−28.4 ± 1.6 mV], which offers enough electrostatic repulsion for colloidal stabilization and comparable to previously developed bio-synthesized iron oxide nanoparticles (Jawed et al., 2023; Ergin et al., 2025; Sathiyabama et al., 2024).

The consistency of the identification of α-Fe_2_O_3_ (hematite) with the JCPDS file (index 33-0664) and the VSM data (near-zero remanence and Ms = 1.84 emu/g) is sufficient to rule out magnetite Fe3O4 as the phase of synthesis. The critical difference is that hematite and magnetite have different crystal structures: rhombohedral and cubic spinel respectively; different magnetic properties: weakly magnetic and strongly ferrimagnetic; different band gap: 2.1 eV for α-Fe_2_O_3_ vs. 0.1 eV for Fe3O4; and different photocatalytic mechanisms: α-Fe_2_O_3_ via electron surface-trapped states while Fe3O4 via surface defects.

The phytochemicals from the Eucalyptus extract are weak reducing agents that reduce the Fe^3^+ ions to α-Fe_2_O_3_, but not to the reduced state Fe_2_IrrFe with Fe^2+^/Fe^3+^ that is necessary for the formation of Fe_3_O_4_ (Kiwumulo et al., 2022). The mildly alkaline synthesis conditions, pH 9, thermodynamically favor precipitation of hematite in this case and this mechanistic hypothesis is consistent with those conditions (Naz et al., 2019; Vinayagam et al., 2020).

Comparative FTIR study confirmed the presence of Phyto molecule mediated capping as the existence of O-H (~3,402 cm^−1^), C-H (~2,920 cm^−1^) and C-O (~1,637 cm^−1^) bands in the extract spectrum is also present in the nanoparticle spectrum. The surface iron atoms are typically oxygen atoms that coordinate with the phenolic -OH of these molecules to provide a layer of organic protection that also prevents iron oxide agglomeration and improves biocompatibility as observed from FTIR studies in the synthesis of iron oxide with plants (Kanase et al., 2020; Zambri et al., 2019; Begum et al., 2025).

The photocatalytic degradation of RhB (~99% in 340 min; *k* = 0.0079 min^−1^) represents strong performance attributable to the visible-light-active bandgap of α-Fe_2_O_3_ (~2.1 eV). The pseudo-first-order kinetics indicate that the photocatalytic rate is proportional to dye concentration throughout the reaction, as expected when catalyst active sites are in excess relative to substrate (Manohar et al., 2021; Madima et al., 2022; Noukelag et al., 2021). The control experiments confirmed that photocatalysis (and not mere adsorption or direct photolysis) is responsible for >85% of the observed degradation. We hypothesize that the organic phytomolecular capping layer on the nanoparticle surface, in addition to stabilizing the colloid, may provide additional electron donor sites that enhance charge separation and reduce electron-hole recombination—a synergistic effect previously proposed for plant-extract-mediated photocatalysts (Saied et al., 2022; Alshawwa et al., 2022). The decrease in performance above 60 mg catalyst loading is mechanistically explained by the Beer-Lambert attenuation of incident photons in increasingly opaque suspensions (“inner filter effect”), which outweighs the benefit of additional active sites (Rostamizadeh et al., 2020).

The antioxidant activity (70% FL reduction at 0.5 mg/ml) is verified as genuine antioxidant activity rather than a cytotoxic artifact: MTT cell viability data confirmed ≥87.4% HDPC cell viability at all tested concentrations, excluding cell death as the explanation for reduced DCF fluorescence. We hypothesize two co-operative mechanisms: (i) direct ROS scavenging by the Fe_2_O_3_ nanoparticle surface through a Fenton-reverse reaction (Fe^3+^ + •OH → Fe^2+^ + H_2_O; •OH consumed rather than generated, in contrast to the oxidative Fenton chemistry operating under photocatalytic conditions); and (ii) antioxidant activity contributed by the adsorbed phytomolecules (polyphenols, flavonoids), as suggested by the non-trivial antioxidant effect of the plant extract alone control (22.4% FL reduction). The comparable efficacy to ascorbic acid at the highest nanoparticle dose supports potential biomedical utility (Lee et al., 2023; Canaparo et al., 2020; Montiel Schneider et al., 2022).

Antibacterial mechanism hypothesis-driven analysis: The superior susceptibility of *S. aureus* (Gram-positive; MIC 0.25 mg/ml) compared to *E. coli* and *B. subtilis* (MIC 0.50 mg/ml) is mechanistically rationalized by the absence of the outer membrane permeability barrier in Gram-positive bacteria. We propose a two-pathway antibacterial mechanism: (i) ROS-mediated oxidative damage to cellular macromolecules (DNA, proteins, lipids), evidenced by the 3.4-fold DCFH-DA fluorescence increase in nanoparticle-treated bacteria; and (ii) direct physical membrane disruption through nanoparticle-membrane contact, as visualized by TEM showing cell wall deformation and cytoplasmic leakage. The MBC/MIC ratio of 2 for all strains confirms bactericidal (not merely bacteriostatic) activity, distinguishing Fe_2_O_3_ NPs from many conventional antibiotics with higher MBC/MIC ratios (Ismail et al., 2015; Gudkov et al., 2021; Shoudho et al., 2024; Gabrielyan et al., 2020).

Recent studies further support the effectiveness of plant-mediated synthesis routes for producing multifunctional iron oxide-based nanomaterials with environmental and biomedical applications (Harini et al., 2024; Zúñiga-Miranda et al., 2023). In recent studies, it has reported that the natural capping effect of phytochemical-rich extracts of these plants on the nanoparticles improves their stability, biocompatibility, and catalytic function (Aswini et al., 2024; Valencia-Islas et al., 2023; Samy et al., 2025). Eucalyptus derived and other Phyto mediated metal-oxide nanomaterials have been found to possess outstanding photocatalytic degradation efficiency toward organic pollutants, better antibacterial activity against pathogenic microorganisms and significant antioxidant properties due to synergistic relationship between the inorganic nanoparticle core and surface bound phytochemicals (Schwan et al., 2023; Abu-Noqta et al., 2019; Shanmugam et al., 2023). These observations are in good agreement with the present results; *Eucalyptus globulus* mediated alpha-Fe_2_O_3_ nanoparticles had high photocatalytic efficiency and also possessed high general spectrum antibacterial activity along with effective ROS-scavanging activity (Sivakami et al., 2025; Elbasuney et al., 2024). The results obtained are consistent with the recent reports that pinpoint the potential of green nanotechnology platform to produce environmentally friendly multifunctional nanomaterials for wastewater treatment, antimicrobial agents, and future biomedical technologies (Nnadozie and Ajibade, 2020; Yusefi et al., 2021; Upadhyay et al., 2016).

In summary, the multifunctional activity of the α-Fe_2_O_3_ nanoparticles synthesized by using the music trees extract can be explained by a single mechanistic model, which involves the high surface area of nanosized particles (8–10 nm) for absorption of photons, for dye adsorption, and for generating ROS upon light irradiation, their natural origin as phytomolecules that can enhance their colloidal stability, biocompatibly and biological activity, and their hematite phase having a bandgap of 2.1 eV which is ideal for visible light driven activity. These results indicate that Eucalyptus Based α-Fe_2_O_3_ is a versatile, reliable, and eco-friendly nanomaterial for environment and biomedical applications.

## 5 Conclusion

In this present study, the eco-friendly synthesis of nanoparticulate α-Fe_2_O_3_ (hematite) has been successfully achieved using the natural extracts of *Eucalyptus globulus* as reducing and capping agent which was confirmed by both XRD (JCPDS 33-0664) and the VSM analysis. The synthesized nanoparticles are quasi-spherical (SEM size: ~10.84 ± 5.22 nm and size by Scherrer equation: ~8 nm) and moderately stable in colloidal suspension as indicated by the zeta potential of −28.4 mV ± 1.6 and PDI value of 0.241. The persistence of O-H, C-H and C-O bands on the surface of the nanoparticles on the FTIR spectra obtained after comparative analysis confirmed the capping by Phyto molecules. The nanoparticles were effective for complete degradation (99 %) of Rhodamine B after 340 min of visible light (λ ≥ 420 nm) application, under pseudo-first order kinetics (*k* = 0.0079 min^−1^), and the experiment in the absence of nanoparticles showed that photocatalytic degradation is the pre-dominant mechanism and not adsorption or sole photolysis. MTT validated antioxidant activity showed that there was ~70% ROS reduction in viable HDPC cells with ≥87.4% viability, representing genuine antioxidant activity. Bactericidal activity was confirmed, MIC range was evaluated as 0.25–0.50 mg/ml and MBC/MIC ratio was between two and three, for all microorganisms tested [*S. aureus* (ATCC 25923), *E. coli* (ATCC 25922) and *B. subtilis* (ATCC 6633)]. These multifunctional Eucalyptus-derived α-Fe_2_O_3_ nanoparticles have integrated properties that suggest them being a sustainable and promising alternative for environmental remediation and biomedical applications.

## Data Availability

The raw data supporting the conclusions of this article will be made available by the authors, without undue reservation.
